# Implentation of a multiomodal approach to improve hand hygiene in a teaching hospital: change of paradigm

**DOI:** 10.1186/1753-6561-5-S6-P254

**Published:** 2011-06-29

**Authors:** RE Quirós, A Novau, L Fabbro, G Desmery

**Affiliations:** 1 Prevention and Infection Control Department, Hospital Universitario Austral, Pilar, Argentina; 2 Nursing School, Universidad Austral, Pilar, Argentina

## Introduction / objectives

Many factors have been involved with low compliance of hand hygiene strategy such as the lack of resources and cultural barriers. In order to improve the level of adherence, a comprehensive program was implemented since Oct 09.

The aim of this study was to evaluate the impact of a multimodal approach to increase the compliance to hand hygiene among health personnel of our institution.

## Methods

The program was based on the concept that “hand hygiene is everyone's commitment” (paradigm change). A multimodal strategy was implemented using WHO recommendations. Alcohol-gel was used as universal method for hand hygiene.Measurements of adherence were carried out identifying the 5 WHO’s opportunities through five cross-sectional studies. The results of each study were shown through a graphic chart to each hospital area in comparison with the rest of institution. Data from Oct 09 were compared with those of Oct’10.

## Results

A total of 4618 observations were performed. The overall adherence to hand hygiene increased from 46.3% to 59.9% (diff: 13.6%, 95% CI 7.7% to 19.5 %, p <0.001). Stratified by type of professional, both nurses and doctors increased their level of adherence (52.1 vs 68.7% [diff: 16.6%, 95% CI 8.3% to 24.9%, p <0.001], 41.6% vs 57.9% [diff: 16.3%, 95% CI 6.6% to 26.0%, p <0.01], respectively). Higher levels of adherence were observed in intensive care units (77%) in comparison with general wards (38%). The consumption of alcohol-gel increased from 49.9 liters per ‰pt-days to 76.5 liters per ‰pt-days (diff: 26.6 ‰, 95% CI 18.4 ‰ to 34.7 ‰, p <0.01).

## Conclusion

The implementation of a multimodal approach in our institution increased the adherence to hand hygiene among health personnel. The feed-back to each hospital area may have helped to raise the level of compliance.

## Disclosure of interest

None declared.

